# Phonological Neighborhood Density and Type Modulate Visual Recognition of Mandarin Chinese: Evidence from Monosyllabic Words

**DOI:** 10.3390/brainsci15121304

**Published:** 2025-12-02

**Authors:** Zhongyan Jiao, Xianhui Zhou, Wenjun Chen

**Affiliations:** 1 School of Foreign Languages, Ningbo University of Technology, Ningbo 315211, China; jiaozhongyan@nbut.edu.cn; 2 School of Humanities and Arts, Ningbo University of Technology, Ningbo 315211, China

**Keywords:** phonological neighborhood, P200, N400, visual word recognition, mandarin

## Abstract

Background: Examining the influence of phonological neighborhoods on the early stages of visual word recognition provides insights into the architecture and dynamics of lexical representation and processing. Methods: Using event-related potentials (ERPs), this investigation explored how phonological neighborhood density (PND; large vs. small) and type (PNT; tone-edit vs. constituent-edit neighbors) influence the recognition of monosyllabic words in Mandarin Chinese. Participants engaged in a priming paradigm combined with a visual lexical decision task. Results: Behavioral data demonstrated the main effect of PNT: words with tone-edit neighbors produced greater processing inhibition compared to those with constituent-edit neighbors. ERP results revealed that large PND enhanced the P200 amplitude, a frontal-mediated effect that was particularly pronounced for tone-edit neighbors. This early differentiation subsequently propelled a stronger N400 response to tone-edit neighbors, culminating in a significant interaction between PND and PNT during the N400 window. Conclusions: These findings support a cascaded competition model: early PND assessment (P200), enhanced for tone neighbors, amplifies their later N400 conflict. This neural mechanism elucidates the hierarchical organization of phonological processing in Chinese monosyllabic words, thereby clarifying a core component which underpins the recognition of more complex words in Mandarin.

## 1. Introduction

Research on silent reading indicates that phonological codes are rapidly and automatically activated during visual word recognition [1,2,3]. Compelling evidence supporting phonology’s role in silent reading emerges from studies examining the influence of phonological neighbors during written word processing [4,5,6]. In alphabetic scripts, phonological neighbors (PNs) denote lexical items that share the same number of phonemes but differ by a single phoneme [7]. The quantity of PNs that a word possesses is termed phonological neighborhood density (PND), a variable demonstrated to significantly influence word recognition across multiple languages [8,9,10].

The distributed framework suggests that the phonological neighborhood effect occurs because a word and its phonological neighbors share many representational units. During lexical access, these shared units become co-activated when the recognition system processes phonologically similar words. In visual word recognition, this co-activation results in faster responses for words with large PND [4,5,11,12]. Yates et al. [11] employed visual lexical decision tasks with Event-related potential (ERP) recordings to manipulate PND. Their behavioral results demonstrated significantly faster reaction times for words from dense phonological neighborhoods compared to sparse neighborhoods. However, the ERP data showed greater N400 amplitudes for words in sparse neighborhoods. The authors proposed that lexical decisions for words with large PND may depend primarily on phonological activation, while words with small PND require additional semantic processing due to weaker phonological representations, as indicated by the enhanced N400.

Notably, most previous studies on PND effects have examined alphabetic languages (e.g., English). However, the phenomenon becomes more intricate in logographic systems like Mandarin Chinese. The Mandarin syllable inventory is notably compact. Syllables follow a simple CGVX structure—comprising an initial consonant (C), a glide (G), a vowel (V), a final consonant (X), and a suprasegmental tone [13]. For instance, the word 宽 (kuan1, width) contains /k/, /u/, /a/, /n/, and tone /1/. These syllables may also be divided into larger constituents [13], for example, as CG_VX (ku_an) or C_GVX (k_uan). A growing body of research demonstrates that segmental and tonal information serve distinct roles in visual word recognition [14], spoken word recognition [15,16,17,18], and word production [19,20]. ERP studies further support a functional dissociation between tonal and segmental processing in Mandarin word recognition [21,22]. Ho et al. [22] observed that tonal violations elicited stronger P200/N400 responses than segmental violations, suggesting tones’ critical role in lexical access. These findings indicate that phonological neighborhoods in Mandarin may exert influence in a more complex, graded manner compared to alphabetic languages.

Based on findings regarding the distinct roles of segments and tones, researchers investigated the relationship between various phonological neighborhood density metrics and lexical retrieval reaction times individually, aiming to identify the optimal neighborhood measure. Using an auditory shadowing task, Neergaard & Huang [23] discovered an inhibitory effect of PND, with measures incorporating both segments and tones (e.g., C_V_C_T and C_G_V_X_T) showing the strongest explanatory power. Yao and Sharma [24], using the phonological neighborhood database established by Neergaard et al. [25], extended this finding in an auditory lexical decision task, similarly revealing inhibitory effects under the C_G_V_X_T schema. Neergaard et al. [13] determined that, in visual lexical decision tasks, an unsegmented syllable-plus-tone schema (CGVX_T) best explained the inhibitory PND effect. This outcome contrasted with their earlier auditory modality findings, which favored a segmented C_V_C_T structure [26]—suggesting task-dependent optimal phonological representation.

Recently, Li et al. [14] proposed a graded model of phonological neighbors in Mandarin Chinese. They classified phonological neighbors into three types: constituent-edit neighbors (following CG_VX or C_GVX schema, as in kuan1 vs. tan1), phoneme-edit neighbors (following the C_G_V_X schema, as in kuan1 vs. tuan1), and tone-edit neighbors (following the CGVX_T schema, as in kuan1 vs. kuan3). Based on experimental findings from three lexical retrieval tasks (word naming, visual lexical decision, and picture naming), the study revealed distinct mechanisms through which different types of phonological neighbors influence lexical retrieval: tone-edit neighbors trigger strong activation and inhibition, manifesting as lexical competition, whereas constituent-edit neighbors facilitate word recognition via weaker activation. This study advances beyond previous research seeking a “single optimal” neighborhood metric by establishing a graded typological framework that clarifies the differential roles of various phonological neighbor types, providing a novel theoretical perspective for understanding early phonological processes in Chinese lexical recognition. It is critical to note that this framework, and the operationalization of PND and PNT, is built upon the phonological structure of monosyllabic units.

Critically, the distinction between tone-edit neighbors and constituent-edit neighbors reported by Li et al. [14] can be explained within a distributed connectionist framework of lexical representation. In such models (e.g., the TRACE model [27] or PDP models [28]), lexical representations emerge as distributed activation patterns across multiple hierarchically organized levels that interact dynamically. Within this architecture, the two neighbor types map onto different representational tiers: tone-edit neighbors differ at the suprasegmental level, when processing a target word (e.g., kuan1), its tone-edit neighbor (e.g., kuan3) generates substantial co-activation and subsequent competition at the whole-word level, as both share identical segmental inputs but activate conflicting tonal representations. In contrast, constituent-edit neighbors differ at the sublexical level (e.g., kuan1 vs. tan1), exhibiting only partial overlap at the sublexical tie, this partial feature sharing likely produces weaker and more diffuse co-activation. This dissociation suggests separable suprasegmental and sublexical levels within an integrated distributed network for morpheme processing. Based on this theoretical account, we designed the present ERP experiment on monosyllabic word recognition, hypothesizing that these two neighbor types would elicit distinct neural signatures reflecting the differing temporal dynamics and neural generators associated with suprasegmental (tonal) and sublexical (segmental) processing at the morpheme level.

Building upon Li et al.’s [14] framework, this study employed a visual lexical decision task with monosyllabic Chinese words. It manipulated tone-edit and constituent-edit neighbors as operationalizations of phonological neighborhood topology (PNT), resulting in a 2 (PND: large vs. small) × 2 (PNT: tone-edit neighbor vs. constituent-edit neighbor) experimental design. A priming paradigm was implemented to control for potential confounding influences from visually or phonologically similar characters on task performance [29]. Based on previous research, a stimulus onset asynchrony (SOA) of 60 ms was selected, which was expected to capture phonological priming but not semantic priming (Perfetti et al. [30] reported graphic effects at 43 ms SOA, phonological effects at 58 ms, and semantic effects at 85 ms). To minimize interference from the form, frequency, and semantic properties of monosyllabic character primes, Pinyin was utilized as the prime format.

The analysis focused on the P200 and N400 components. Convergent evidence from studies on Chinese character processing indicates that the P200 component functions as an indicator of early phonological processing [31,32,33]. Additionally, ERP studies have demonstrated that both segmental and tonal information are detected within a 170–280 ms time window [21,22]. Research across auditory and visual modalities consistently shows that tonal violations modulate P200 amplitude, supporting the perspective that this component reflects early categorical perception of phonological information. The N400 component serves as a sensitive index of semantic processing difficulty, reflecting costs associated with lexical-semantic access or integration [34]. Based on this evidence, two hypotheses were examined regarding the relative roles of PND and PNT in monosyllabic Chinese word reading. First, tone-edit neighbors were expected to generate significantly larger P200 and N400 amplitudes than constituent-edit neighbors, reflecting enhanced morpheme-level competition due to greater phonological similarity. Second, large PND conditions were predicted to amplify these ERP effects, indicating density-dependent modulation of phonological activation.

## 2. Materials and Methods

### 2.1. Participants

The sample size for this study was determined with reference to the range commonly employed in comparable studies [11,21] to ensure comparability with the existing literature. Therefore, a total of 33 native Mandarin speakers (10 males, 23 females; mean age = 20.5 years, SD = 0.9) were recruited. All participants were right-handed and had normal or corrected-to-normal vision. Informed consent was obtained prior to participation, and all participants received compensation for their involvement.

### 2.2. Stimuli and Design

The stimulus materials were selected from the publicly available “Word Neighborhood Database” developed by Li et al. [14]. This database limits its neighborhood calculations to monosyllabic words and contains lexical frequency, PND and PNF calculations for 4706 words. For polyphonic characters, the most frequent pronunciation was selected to ensure disambiguation. Li et al. manually verified all pronunciations against the Xinhua Dictionary to ensure their typicality and normative status.

The calculation of PND followed the method used in Li et al. [14], defined as the number of phonological neighbors of a word. This count was derived by calculating the edit neighbors for a given monosyllable (e.g., kuan1) at the tone- (kuan3), constituent- (tan1), and phoneme-levels (tuan1). To prevent repetitive counting, each word contributed only once as a neighbor to the target word. When a token could be categorized as more than one type of neighbor, priority was assigned to the neighbor type requiring the smallest edit distance in terms of phonological units.

A total of 120 Mandarin monosyllabic words served as critical items (see Supplementary Materials Tables S1 and S2 for details), which were all rigorously screened to have a single dominant pronunciation and core meaning to control for confounds from polyphony or polysemy. The critical items were categorized into two PND conditions: (1) large PND (*n* = 60; M = 213, SD = 23) and (2) small PND (*n* = 60; M = 119, SD = 31). The two groups were matched in stroke count (t(118) = 0.042, *p* = 0.966) and lexical frequency (t(118) = 1.124, *p* = 0.263). It should be noted that, as is inherent in studies employing natural lexicons, a positive correlation exists between phonological neighborhood density and the frequency of phonological neighbors (PNF) in our stimulus set. To account for this potential confound, PNF was included as a covariate in the subsequent statistical analyses.

Detailed stimulus properties are provided in Table 1. Each target word was paired with two prime types. (1) Constituent-edit primes differed from the target in onset or rime while maintaining the same tone (e.g., lüe4-lao4). (2) Tone-edit primes maintained identical segments with the target but varied in tone (e.g., lao2-lao4). The Pinyin length of all primes was strictly controlled. Following Neergaard et al. [13], Pinyin length denotes the number of letters (excluding tone numbers) used to construct an item’s Pinyin spelling. For instance, the Pinyin length of “酪 (lao4, cheese)” is 3. Primes were selected from each target’s legitimate phonological neighbors in the database, prioritizing minimal edit distance. When multiple candidates satisfied the criteria, one was randomly selected. This process generated 240 target-prime test pairs.

To maintain experimental balance, 120 Chinese pseudocharacters were constructed by recombining legal sublexical components from the target characters (see Supplementary Material Table S3 for details). These pseudocharacters: (1) adhered to Chinese orthographic combination rules, (2) matched the structural topology of the target characters (e.g., left-right, top-bottom, or single-unit configurations), (3) demonstrated comparable visual complexity (target characters: 8.83 ± 2.84 strokes; pseudocharacters: 8.68 ± 2.12 strokes), and (4) possessed no semantic or phonological validity (e.g., target: 酪; pseudocharacter: 䣯). Each pseudocharacter was paired with identical prime types as its corresponding target character, generating 240 filler trials.

Within each PND group (large/small), each target character was presented under two priming conditions (constituent-edit and tone-edit) in a within-subjects design. Pseudocharacter trials served exclusively as fillers and were excluded from subsequent analyses.

All stimuli appeared in black text on a white background and were organized into six Latin-square blocks, with each block containing all experimental conditions. Conditions were pseudorandomly counterbalanced and presented in a randomized sequence.

### 2.3. Apparatus and Procedure

**Figure 1:**
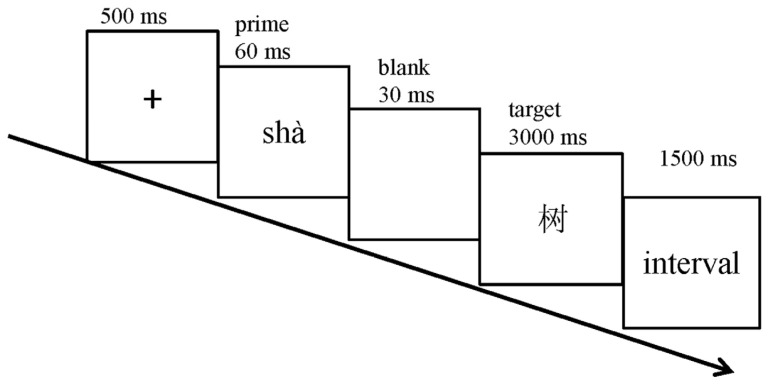
Experimental procedure for a single trial, where the character ‘树’ (meaning ‘tree’) is presented as the target stimulus.

The experiment was implemented using E-Prime 3.0 (Psychology Software Tools) and displayed on a 21-inch LCD monitor. Prime stimuli appeared in 12-point Times New Roman font, while target words were presented as centrally positioned 6 × 6 cm images. Participants were positioned 60 cm from the screen during individual testing sessions. Each trial (see Figure 1) followed a specific sequence: (1) a fixation cross (‘+’) appeared for 500 ms; (2) the prime was displayed for 60 ms, followed by a blank screen for 30 ms; and (3) the target word appeared for 3000 ms. Participants indicated lexical decisions using the ‘J’ key for real words and the ‘F’ key for nonwords. Trials without responses within the 3000 ms window automatically terminated and were recorded as incorrect. Before the main task, participants completed 36 practice trials to familiarize themselves with the procedure. These practice items were excluded from the main analysis. The complete experiment required approximately 40 min.

### 2.4. EEG Recording and Preprocessing

EEG data were recorded using a 32-channel actiCAP system (Brain Products GmbH, Gilching, Germany) with Ag/AgCl active electrodes arranged according to the international 10–20 system. The left mastoid served as the online reference, and electrode impedance was maintained below 5 kΩ. Data preprocessing was conducted using EEGLAB in MATLAB 2018b (MathWorks, Natick, MA, USA). EEG signals were amplified with a 0.05–100 Hz band-pass filter and digitized at 500 Hz. Offline preprocessing comprised re-referencing to the right mastoid and applying a 0.1–30 Hz band-pass filter. EEG epochs were extracted from −200 to 800 ms relative to stimulus onset and baseline-corrected using the −200 to 0 ms window. Noisy channels were corrected through spherical spline interpolation. Artifacts were corrected using Independent Component Analysis (ICA). After computing ICA, the ICLabel toolbox was applied to automatically classify each independent component. Final decisions regarding component rejection were made manually by the experimenter. Specifically, components with a high probability score (>90%) from ICLabel for the categories Eye and Muscle were visually inspected; if confirmed as artifacts, they were excluded from the dataset. Finally, epochs exceeding ±80 µV were rejected. The overall mean trial rejection rate was 7.1%, ranging from 5.8% to 8.2%.

Data from four participants were excluded due to excessive artifacts. Additionally, nonword trials and trials with incorrect behavioral responses were removed from the EEG analysis. The final dataset included 29 participants, with an average of 1616 valid trials per condition retained for subsequent analyses.

## 3. Results

### 3.1. Behavioral Data

#### 3.1.1. Non-Words

Analysis of non-words revealed that participants accurately categorized 90% of the non-words in the lexical decision task, demonstrating reliable engagement in the lexical decision process and successful rejection of non-words.

#### 3.1.2. Real-Words

As shown in Table 2, accuracy rates were uniformly high (range: 97.7–99.3%) and stable across conditions. Therefore, our analyses focused on reaction times (RTs) and ERP data, which are more sensitive indicators of subtle differences in cognitive processing.

The final analysis included data from 29 participants. Correct responses with reaction times (RTs) exceeding 3 standard deviations from the mean RT for each condition were also excluded. In total, 137 data points (approximately 1.9% of the dataset) were removed. The mean RTs and accuracy rates for the experiment are presented in Table 2.

The analysis of RTs was conducted using linear mixed-effects models (LMMs) [35], implemented with the lme4 package [36]. *p*-values were calculated using the lmerTest package [37] in R [38]. The analysis specifically examined correctly responded target words. To determine the optimal model, likelihood-ratio tests compared a model containing fixed effects of PNT, PND, and their interaction against simpler nested models. To control for potential confounding by PNF (PNF values calculated by Li et al. [14]), PNF was included as a covariate in the final model. The model specification was: lmer (logRT ~ PNT × PND + PNF + (1|SUB) + (1|ITEM)).

**Figure 2:**
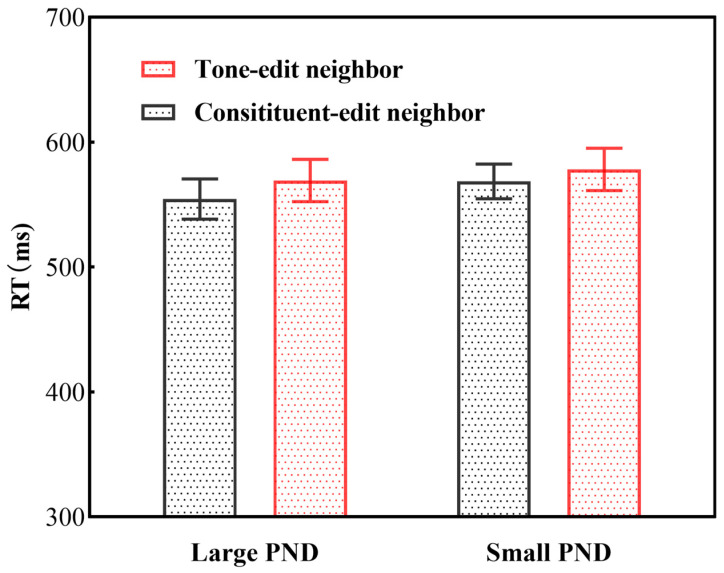
Mean reaction times (RTs) and standard deviations across conditions.

Results indicated a significant main effect of PNT, with longer reaction times for tone-edit neighbors (RT = 572 ms) compared to constituent-edit neighbors (RT = 555 ms), b = −0.0357, SE = 0.0071, t = −5.018, *p* < 0.001 (see Figure 2). No significant main effect of PND was found (b = 0.0044, SE = 0.0078, t(383.8) = 0.567, *p* = 0.571). The interaction between PNT and PND did not reach statistical significance (b = 0.0154, SE = 0.0101, t = 1.524, *p* = 0.128). Crucially, the PNF covariate was not significant (b = 2.058 × 10^−7^, SE = 2.284 × 10^−7^, t = 0.901, *p* = 0.369), indicating that the observed effects are independent of neighborhood frequency differences.

### 3.2. ERP Data

Following previous research [11,21,22,31] and examination of the grand average waveform, mean amplitudes of the P200 and N400 components were analyzed. The P200 component was examined within the 200–260 ms post-stimulus window, and the N400 component was analyzed within the 300–500 ms window.

To address potential confounding effects of PNF, a comprehensive analysis of trial-level ERP data was conducted using linear mixed-effects models [35]. For the P200 component, electrodes were grouped into three regions of interest (ROIs): frontal (Fz, F7, F8), central (C3, Cz, C4), and parietal (P3, Pz, P4). For the N400 component, central (C3, Cz, C4) and parietal (P3, Pz, P4) ROIs were selected. Statistical analyses were performed using linear mixed-effects models, with ROI (frontal, central, parietal) included as a fixed-effects factor. Mean amplitudes were computed by averaging across electrodes within each ROI prior to analysis. Model comparison procedures revealed a significant improvement in fit upon inclusion of subject-by-ROI random intercepts (χ^2^(1) = 2150.3, *p* < 0.001), leading to the final model specification: lmer (MeanAmplitude ~ pnd × pnt × Region + PNF_z + (1|Subject) + (1|Subject: ROI) + (1|item). All analyses were performed using the lme4 [36] and lmerTest packages [37] in R [38], employing restricted maximum likelihood (REML) estimation and Satterthwaite’s method for degrees of freedom approximation. ERP results for each component are presented below.

#### 3.2.1. P200 (200–260 ms)

**Figure 3:**
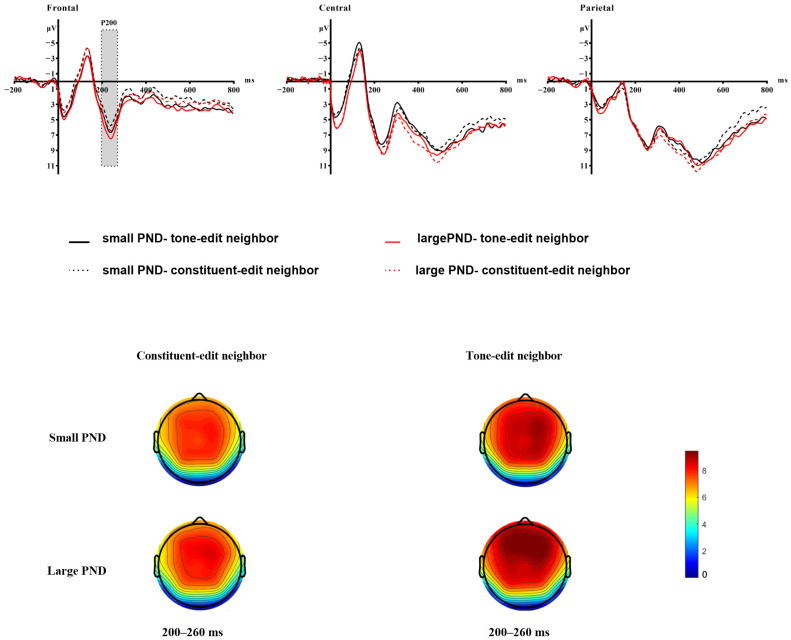
The P200 component during the lexical decision task. This figure displays the average event-related potentials (ERPs) and topographic maps across four conditions in the lexical decision task. (Top) Event-related potential (ERP) waveforms for the P200 time window (200–260 ms), averaged across electrodes within each ROI: Frontal region (Fz, F7, F8), Central region (C3, Cz, C4) and Parietal region (P3, Pz, P4). (Bottom) The topographic maps illustrate the voltage distribution across the scalp during the 200–260 ms time window relative to baseline, with the color scale ranging from 0 to +8 μV.

For the P200 component, results revealed a significant main effect of PND, b = −0.624, SE = 0.186, t = −3.354, *p* < 0.001, indicating that the large PND condition elicited enhanced (more positive) P200 amplitudes relative to the small PND condition (see Figure 3). A significant three-way interaction between PND, PNT, and ROI was observed, particularly for the Frontal region, b = −0.794, SE = 0.370, t = −2.146, *p* = 0.032. Additionally, a significant two-way interaction between PNT and Frontal region emerged, b = 0.691, SE = 0.260, t = 2.655, *p* = 0.008. No other main effects or interactions reached significance (all ps > 0.05). Simple effects analysis within the Frontal region showed that under tone-edit condition, large PND elicited significantly larger P200 amplitudes than small PND, b = 1.214, SE = 0.187, z = 6.507, *p* < 0.001, while under large PND condition, tone-edit neighbors generated larger amplitudes than constituent -edit neighbors, b = −0.626, SE = 0.185, z = −3.392, *p* = 0.004 (see Figure 3). Critically, the PNF covariate was not significant, b = −0.112, SE = 0.084, t = −1.342, *p* = 0.181, indicating that the observed effects were not confounded by phonological neighborhood frequency.

#### 3.2.2. N400 (300–500 ms)

**Figure 4:**
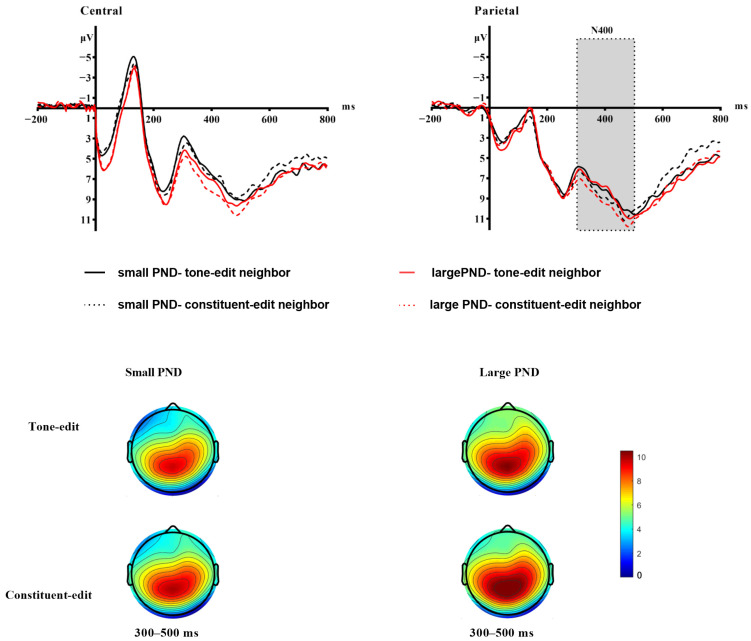
The N400 component during the lexical decision task. This figure displays the average event-related potentials (ERPs) and topographic maps across four conditions in the lexical decision task. (Top) Event-related potential (ERP) waveforms for the N400 time window (300–500 ms), averaged across electrodes within each ROI: Central region (C3, Cz, C4) and Parietal region (P3, Pz, P4). (Bottom) The topographic maps illustrate the voltage distribution across the scalp during the 300–500 ms time window relative to baseline, with the color scale ranging from 0 to +10 μV.

Results revealed a significant main effect of PND, b = 1.575, SE = 0.177, t = 8.919, *p* < 0.001. A significant main effect of PNT was also observed, b = −0.445, SE = 0.177, t = −2.508, *p* = 0.012. Crucially, a significant two-way interaction between PND and PNT emerged, b = −0.669, SE = 0.250, t = −2.679, *p* = 0.007. Simple effects analysis revealed that tone-edit neighbors elicited more negative N400 amplitudes than constituent-edit neighbors at both levels of PND, but was substantially stronger under large PND condition (*p* < 0.001) than under small PND condition (*p* = 0.026). ROI analysis showed a significant main effect, b = −2.139, SE = 0.584, t = −3.665, *p* < 0.001, with Parietal region exhibiting more negative N400 amplitudes than Central region (see Figure 4). Finally, the PNF covariate was not significant, b = −0.131, SE = 0.083, t = −1.577, *p* = 0.116, indicating that the observed effects were not confounded by phonological neighborhood frequency.

## 4. Discussion

This study investigated the electrophysiological correlates of phonological neighborhood density (PND) and type (PNT) during Chinese monosyllabic visual word recognition. Behavioral data demonstrated a main effect of PNT, with words possessing tone-edit neighbors yielding longer reaction times than those with constituent-edit neighbors. ERP results revealed distinct hierarchical stages of processing: an early P200 modulation by PND, followed by later N400 effects where PNT was qualified by its interaction with PND. This pattern indicates that density and type exert differential influences at separate stages of morpheme-level processing.

The dissociation between behavioral reaction times and ERP measures offers crucial insight into the dynamics of Chinese monosyllabic lexical recognition. Behaviorally, only a main effect of PNT emerged, with no effect of PND or a PND × PNT interaction. In contrast, ERP data revealed a significant main effect of PND during the P200 time window. This pattern suggests that while the influence of PND is not apparent in the final behavioral output, it plays a significant role in early neural processing. The temporal resolution of ERP enabled observation of large PND generally amplifying the P200 neural response approximately 200 ms after stimulus onset, reflecting early competition among co-activated representations in a dense phonological network. Behavioral reaction time, as an endpoint measure integrating multiple subsequent stages (e.g., lexical selection, semantic integration, response execution), allows later mechanisms to compensate for the early competition modulated by PND, leaving only the independent effect of PNT observable at the behavioral level. This dissociation highlights the distinct advantage of neurophysiological measures in revealing transient, underlying processing mechanisms that remain invisible to behavior alone.

The significantly larger P200 amplitude under large PND underscores the role of PND in early phonological processing. We interpret this enhancement as reflecting rapid and broad phonological co-activation at the syllable level. According to distributed models of word recognition [39,40], a word from a dense phonological neighborhood shares features with many neighbors, leading to rapid co-activation of a broad phonological cluster upon stimulus onset. This early, widespread phonological activation is reflected in the enhanced P200. The finding that this effect was particularly pronounced in the Frontal region for tone-edit neighbors under large PND conditions (as indicated by the significant three-way interaction) suggests that anterior brain regions may be particularly sensitive to the demands of processing dense phonological networks, especially when they contain highly similar competitors.

Alternative interpretations, though less supported by the current data, merit consideration. The P200 enhancement under large PND could be facilitative, reflecting strengthened feedback from broad phonological co-activation to orthographic representations, thereby enhancing early visual-linguistic integration [41]. However, the overall pattern of results—specifically, that the early, density-driven P200 enhancement is followed by a magnified competitive effect from tone-edit neighbors in the N400 window—aligns more parsimoniously with the view that the P200 reflects increased initial processing effort or the scope of competition within a dense phonological neighborhood. This early effort subsequently culminates in the more pronounced lexico-semantic competition observed later in time. Future research is warranted to systematically dissociate these possibilities.

N400 serves as a reliable marker of semantic processing and contextual integration. Tone-edit neighbors elicited significantly greater N400 amplitudes compared to constituent-edit neighbors. Crucially, this effect was modulated by phonological neighborhood density, as evidenced by the significant PND × PNT interaction. Simple effects analysis confirmed that the interference from tone-edit neighbors was substantially stronger under large PND conditions than under small PND conditions. This pattern aligns closely with predictions from connectionist frameworks. Competition from tone-edit neighbors, which share complete segmental overlap with the target, requires strong mutual inhibition at the lexical node level for resolution. When this competition occurs within a dense neighborhood (large PND), the number of co-activated competing candidates is greater, thereby amplifying the selection difficulty and resulting in a greatly enhanced N400 amplitude. These findings emphasize the crucial role of tone in Chinese monosyllabic semantic access: tonal violations introduce interference during character comprehension. While segmental phonological information undergoes early activation (as reflected in the P200 component), tonal violations disrupt subsequent phonology-to-semantics mapping, requiring greater neural resources for successful semantic resolution.

Empirical support for the role of tone in semantic processing has emerged from multiple experimental paradigms. Ho et al. [22] employed a cross-modal sentence paradigm and found that tonal violations (TV) elicited the most negative amplitudes during the 400–500 ms time window (the typical N400 time range), with significantly stronger effects than those induced by onset violations (OV) or syllable violations (SV), highlighting the decisive influence of tone on lexical prediction. Similarly, Brown-Schmidt and Canseco-Gonzalez [42] reported robust N400 responses to tonal violations during sentence comprehension tasks, whereas segmental violations produced comparatively weaker semantic interference. These findings confirm that in Mandarin, tones function not merely as phonological features but as obligatory cues for lexical-semantic integration.

The present study behaviorally corroborates Neergaard et al.’s [13] finding that unsegmented syllable + tone schema (CGVX_T) plays a dominant role in visual word recognition of Chinese monosyllables, while also elucidating its underlying temporal dynamics through ERP measures. Our data demonstrate a cascaded process: during the early perceptual stage (200–260 ms), large phonological neighborhood density elicited larger P200 amplitudes, indicating that early phonological processing is modulated by the overall scale of phonological co-activation. Subsequently, in the lexical-semantic integration stage (300–500 ms), the type of closest competitor and its interaction with the density of the neighborhood jointly determined the effort required for semantic access, as reflected in the N400. This temporal fractionation provides a more nuanced neurocognitive account of the unsegmented schema’s influence. These findings contrast with Neergaard et al.’s [25] auditory results, which supported a segmented schema (C_V_C_T), collectively indicating a modality-dependent nature of phonological processing in Chines: visual word recognition relies more on unsegmented syllable representations, whereas auditory processing favors sequential segmental analysis. The root of this divergence lies in the fundamental nature of the Chinese character as a basic grapho-phonological unit, which maps directly onto a holistic syllable during visual recognition. In contrast, auditory processing is necessarily bound to a sequential parsing of the temporal speech stream.

Li et al. [14] did not report significant inhibitory effects of tone neighbors in their lexical decision task. The present study, however, provides clear evidence for this inhibition both behaviorally, through longer reaction times for tone-edit neighbors, and electrophysiologically, as reflected in the enhanced N400 amplitude for these items. This discrepancy may arise from methodological differences: while Li et al. [14] used a simple lexical decision task, our study employed a primed lexical decision paradigm, introducing an early phonological activation stage. This priming likely alters processing dynamics by enabling full activation of phonological representations—particularly tonal information—at an early stage, thereby allowing competition arising from tonal conflict to propagate more clearly into subsequent behavioral responses and neural activity.

Collectively, our ERP data provide initial, support for Li et al.’s [14] graded neighborhood model at the monosyllabic level, and more importantly, successfully delineate the temporal dynamics of these distinctions within a cascaded processing framework. it captures fundamental functional differences that unfold over time. The early P200 component, particularly over frontal regions, was primarily sensitive to the scale of phonological competition (PND), a process that was differentially enhanced for tone-edit neighbors. This early, density-sensitive differentiation then set the stage for the full manifestation of suprasegmental competition during later lexico-semantic access, as captured by the robust PNT effect and its interaction with PND in the N400 component.

## 5. Limitations and Future Directions

Although this study offers valuable electrophysiological insights into the temporal dynamics of phonological neighborhood effects during monosyllabic Chinese word recognition, several conceptual and methodological constraints merit consideration. By limiting phonological neighborhood calculations exclusively to monosyllabic units, we adopted a simplified representation of Chinese morphological structure, which inherently depends on disyllabic compounding and morphosyllabic organization. This approach may not fully capture the semantically grounded networks underlying the mental lexicon, particularly for disyllabic words. Furthermore, the phonological neighborhood construct—originally developed for alphabetic languages—requires careful evaluation when applied to Chinese, given fundamental differences in lexical processing. Consequently, these conceptual and methodological constraints suggest that results derived from monosyllabic metrics should be extrapolated with caution to natural mandarin Chinese representation and processing.

Finally, building on the core linguistic characteristic that the Chinese lexicon is predominantly composed of highly productive disyllabic compounds, for instance, the monosyllabic word”dian4” (电, electricity) frequently occurs in disyllabic words such as “dian4ying3” (电影, movie), “dian4hua4” (电话, telephone), and “shan3dian4” (闪电, lightning), future research should advocate a shift in metric paradigms from a “monosyllabic-node” focus to a “whole-word network” approach. Specifically, it is essential to adopt integrated phonology-semantics metrics based on disyllabic words to systematically measure whole-word phonological neighborhood density, semantic association strength, and lexical frequency distribution. Doing so would thereby allow a rigorous examination of whether the time-course dynamics of phonological competition observed at the monosyllabic level in this study (e.g., the P200–N400 effects) can be generalized to disyllabic word processing, which better reflects natural language use. Such a direction will ultimately contribute to constructing a more ecologically valid cognitive model of the Chinese lexicon.

## 6. Conclusions

Our findings provide electrophysiological support for a graded neighborhood organization in the Chinese Monosyllabic Words. They demonstrate a temporal dissociation in the processing of phonological neighborhood properties: an early assessment of neighborhood density (PND) is observed in the P200 component, which is particularly sensitive to tone neighbors, and this modulates the strength of later lexico-semantic competition from these neighbors, as reflected in the N400. Together, these results clarify the specific neural mechanisms governing early, morpheme-based phonological processing in Chinese, establishing a foundational framework for understanding the architecture of the Mandarin mental lexicon.

## Figures and Tables

**Table 1:** Descriptive statistics for two types of critical characters and their paired prime characters.

		Large PND	Small PND
Sample character		lao4-酪 (cheese)	gou3-狗 (dog)
LogCHR		3.46 (0.65)	3.57 (0.52)
Strokes		8.63 (2.01)	8.65 (2.29)
PND		213.12 (23.14)	119.23 (31.78) ***
Neighborhood type	tone-edit	lao2	gou4
	constituent-edit	lüe4	gai3
Pinyin length	tone-edit	3.78 (0.61)	3.62 (0.61)
	constituent-edit	3.73 (0.69)	3.93 (0.64)

Note: All stimuli were selected from the word neighborhood database created by Li et al. [14]. All psycholinguistic variables, including LogCHR and PND values, were calculated by the authors. Standard deviations are shown in parentheses. *** *p* < 0.001.

**Table 2:** Mean reaction times (ms) and accuracy rates (%) for targets (Large PND and Small PND) primed by phonological neighbors (constituent-edit and tone-edit).

	Consitituent_Edit	Tone_Edit Neighbor
PND	(e.g., lüe4-酪) (cheese)	(e.g., lao2-酪) (cheese)
Large	551 (0.993)	572 (0.986)
Small	558 (0.984)	571 (0.977)

Note: Accuracy rates are presented in parentheses.

## Data Availability

The data that support the findings of this study are available from the corresponding author upon reasonable request. The data are not publicly available to protect the privacy of research participants.

## Supplementary Materials

The following supporting information can be downloaded at: https://www.mdpi.com/article/10.3390/brainsci15121304/s1, Table S1: Full List of real word Stimulus; Table S2: Psycholinguistic Variables of Stimulus Materials; Table S3: Full List of Pseudocharacter Stimulus.
