# Supplementary material for: Phonological Neighborhood Density and Type Modulate Visual Recognition of Mandarin Chinese: Evidence from Monosyllabic Words
Source: Brain Sci. 2025 Dec 2;15(12):1304. doi: 10.3390/brainsci15121304 (PMC12730931; doi:10.3390/brainsci15121304)
Supplement: Supplementary file 1 [file brainsci-15-01304-s001.zip › Supplementary Table S1: Full List of real word Stimulus.pdf]

Full List of real word Stimulus

Large PND group			Small PND group		
Tone- prime	Constituent- prime	Target	Tone- prime	Constituent- prime	Target
jia3	jue1	加	liang2	huang3	两
zhan1	zhui4	战	kan4	kua3	砍
jie2	xue4	界	cheng4	chuan1	称
shui3	shen4	睡	shen4	shuo1	伸
ban3	bei4	半	gou4	gai3	狗
huan1	shan4	换	tuo2	tou1	脱
duan1	dui4	段	rao4	rui3	扰
liao2	ling4	料	zai1	zuo4	载
luan3	zhan4	乱	zuo3	zei2	昨
jia1	cha4	架	tong3	tuan2	佟
zhuan3	zheng1	专	yang4	qiang2	佯
jie3	jun4	介	heng4	huan1	亨
liao4	ling2	疗	nao2	nei3	脑
dan1	dui4	诞	pang4	piao2	庞
zhao4	zhen1	招	zang1	zuan4	葬
jie1	jun4	借	han4	huo3	喊
shan1	shui4	善	shan4	shuo1	珊
ban1	bie4	拌	jin4	jue1	筋
huan4	chan2	环	dao4	dun3	倒
lao3	lou2	劳	rou4	ren2	柔
jiao1	jing4	叫	zhang3	xiang1	章
xuan3	xing1	宣	hun2	hou1	昏
lan2	lue4	烂	chang1	sang4	倡
lan3	lei2	蓝	dian4	ding3	典
lao1	liu2	牢	cha1	xia2	茬
ban3	bei4	扮	chan3	chui1	掺
liang4	liao2	量	jin1	xun4	劲
lang4	luan2	廊	mao2	miu4	冒
jiao3	jing1	骄	chen4	chui2	尘
juan4	zhan1	捐	qian4	qing2	前
lang4	luan2	狼	xiu4	xue1	羞
han3	hou2	含	xian1	xing2	闲
huan1	chan4	患	cao3	cen2	曹
zhui1	zhao4	坠	jie4	xue2	竭
guan4	xian1	官	luan4	ling3	卵
jiao3	sao1	胶	xiu1	xun4	秀
dan1	duo4	淡	bian4	bang1	边
zhuan4	kuan1	砖	yuan4	ying2	原
xuan1	jian4	眩	tiao2	tuan2	调
jiao1	jiu4	较	chen4	chui2	臣
lang4	liao2	郎	beng1	bian4	泵
xuan1	qian4	绚	mei2	min3	每
guan3	gang1	倌	zhen1	zhou4	阵
xuan2	xiang1	轩	ting4	tuan1	厅
liang3	huang2	凉	xian1	xing4	羡
luan4	xian2	峦	mao1	men2	矛
jiang2	shuang1	姜	kai1	kua3	凯
juan4	chuan1	娟	qian2	qing4	欠

wen2	wai4	问	ming2	miao4	命
mian3	ming4	面	geng3	guan1	更
liang4	chang2	良	sui4	san2	随
ban3	bei4	伴	cai4	cen3	采
guan1	xian4	冠	pei4	pin2	陪
luan3	qian2	孪	xiang4	chang3	响
juan1	bian3	卷	fan4	fei3	反
xuan1	jian4	铉	ting2	tuan1	听
bei1	ban4	被	suo3	sou1	唆
lao2	lue4	酪	shao1	shua3	少
guan3	dian1	观	qiao3	qing1	跷
luan4	pian2	鸾	zhan4	zhuo1	詹
