# Supplementary material for: Phonological Neighborhood Density and Type Modulate Visual Recognition of Mandarin Chinese: Evidence from Monosyllabic Words
Source: Brain Sci. 2025 Dec 2;15(12):1304. doi: 10.3390/brainsci15121304 (PMC12730931; doi:10.3390/brainsci15121304)
Supplement: Supplementary file 1 [file brainsci-15-01304-s001.zip › Supplementary Table S2: Psycholinguistic Variables of Stimulus Materials.pdf]

Psycholinguistic Variables of Stimulus Materials

item	PND Group	pinyin	Strokes	PND	logCHR	Tone-edit neighbor	Pinyin length (Tone-edit neighbor)	Constituent-edit neighbor	Pinyin length (Constituent-edit neighbor)
加	large	jia1	5	209	4.5816	jia3	3	jue1	3
战	large	zhan4	9	224	4.1674	zhan1	4	zhui4	4
界	large	jie4	9	204	4.004	jie2	3	xue4	3
睡	large	shui4	13	204	4.0643	shui3	4	shen4	4
半	large	ban4	5	214	4.0378	ban3	3	bei4	3
换	large	huan4	10	201	3.9895	huan1	4	shan4	4
段	large	duan4	9	223	3.9012	duan1	4	dui4	3
料	large	liao4	10	207	3.8308	liao2	4	ling4	4
乱	large	luan4	7	240	3.8235	luan3	4	zhan4	4
架	large	jia4	9	205	3.7043	jia1	3	cha4	3
专	large	zhuan1	4	207	3.7872	zhuan3	5	zheng1	5
介	large	jie4	4	204	3.7863	jie3	3	jun4	3
疗	large	liao2	7	214	3.6804	liao4	4	ling2	4
诞	large	dan4	8	217	3.7361	dan1	3	dui4	3
招	large	zhao1	8	201	3.7277	zhao4	4	zhen1	4
借	large	jie4	10	204	3.7087	jie1	3	jun4	3
善	large	shan4	12	219	3.7068	shan1	4	shui4	4
拌	large	ban4	7	214	2.5011	ban1	3	bie4	3
环	large	huan2	8	200	3.6856	huan4	4	chan2	4
劳	large	lao2	7	228	3.6604	lao3	3	lou2	3
叫	large	jiao4	10	229	4.6156	jiao1	4	jing4	4
宣	large	xuan1	9	216	3.5948	xuan3	4	xing1	4
烂	large	lan4	9	247	3.5948	lan2	3	lue4	3
蓝	large	lan2	13	233	3.5519	lan3	3	lei2	3
牢	large	lao2	7	228	3.5181	lao1	3	liu2	3

item	PND Group	pinyin	Strokes	PND	logCHR	Tone-edit neighbor	Pinyin length (Tone-edit neighbor)	Constituent-edit neighbor	Pinyin length (Constituent-edit neighbor)
扮	large	ban4	7	214	3.4881	ban3	3	bei4	3
量	large	liang2	12	220	3.4836	liang4	5	liao2	4
廊	large	lang2	11	217	3.1209	lang4	4	luan2	4
骄	large	jiao1	9	224	3.3371	jiao3	4	jing1	4
捐	large	juan1	10	234	3.2591	juan4	4	zhan1	3
狼	large	lang2	10	217	3.246	lang4	4	luan2	4
含	large	han2	7	200	3.237	han3	3	hou2	3
患	large	huan4	11	201	3.1875	huan1	4	chan4	4
坠	large	zhui4	7	200	3.0191	zhui1	4	zhao4	4
官	large	guan1	6	208	4.3305	guan4	4	xian1	4
胶	large	jiao1	10	224	3.1801	jiao3	4	sao1	3
淡	large	dan4	11	217	3.0133	dan1	3	duo4	3
砖	large	zhuan1	9	207	2.6637	zhuan4	5	kuan1	4
眩	large	xuan4	10	209	2.2355	xuan1	4	jian4	4
较	large	jiao4	10	229	3.6167	jiao1	4	jiu4	3
郎	large	lang2	8	217	3.3555	lang4	4	liao2	4
绚	large	xuan4	9	209	2.6232	xuan1	4	qian4	4
倌	large	guan1	10	208	2.0792	guan3	4	gang1	4
轩	large	xuan1	7	216	2.6021	xuan2	4	xiang1	5
凉	large	liang2	10	220	3.0099	liang3	5	huang2	5
峦	large	luan2	9	246	2.3222	luan4	4	xian2	4
姜	large	jiang1	9	210	2.6637	jiang2	5	shuang1	6
娟	large	juan1	10	234	2.9494	juan4	4	chuan1	5
问	large	wen4	6	89	4.7087	wen2	3	wai4	3
面	large	mian4	9	147	4.6262	mian3	4	ming4	4
良	large	liang2	7	220	3.4836	liang4	5	chang2	5
伴	large	ban4	7	214	3.6861	ban3	3	bei4	3

item	PND Group	pinyin	Strokes	PND	logCHR	Tone-edit neighbor	Pinyin length (Tone-edit neighbor)	Constituent-edit neighbor	Pinyin length (Constituent-edit neighbor)
冠	large	guan4	9	208	3.4767	guan1	4	xian4	4
孪	large	luan2	9	246	2.085	luan3	4	qian2	4
卷	large	juan3	8	243	3.523	juan1	4	bian3	4
铉	large	xuan4	10	209	2.1614	xuan1	4	jian4	4
被	large	bei4	10	179	4.8458	bei1	3	ban4	3
酪	large	lao4	13	205	3.1449	lao2	3	lue4	3
观	large	guan1	6	208	3.9528	guan3	4	dian1	4
鸾	large	luan2	11	246	2.699	luan4	4	pian2	4
两	small	liang3	7	121	3.4079	liang2	5	huang3	5
砍	small	kan3	9	99	3.0792	kan4	3	kua3	3
称	small	cheng1	10	101	3.7916	cheng4	4	chuan1	5
伸	small	shen1	7	123	3.4145	shen4	4	shuo1	4
狗	small	gou3	8	92	4.1454	gou4	3	gai3	3
脱	small	tuo1	11	96	3.9001	tuo2	3	tou1	3
扰	small	rao3	7	104	3.7538	rao4	3	rui3	3
载	small	zai4	10	105	3.3418	zai1	3	zuo4	3
昨	small	zuo2	9	79	4.0027	zuo3	3	zei2	3
佟	small	tong2	7	122	2.9031	tong3	4	tuan2	4
佯	small	yang2	8	128	3.8118	yang4	4	qiang2	5
亨	small	heng1	7	95	3.1998	heng4	4	huan1	4
脑	small	nao3	10	110	4.1441	nao2	3	nei3	3
庞	small	pang2	8	139	3.8733	pang4	4	piao2	4
葬	small	zang4	12	103	3.2984	zang1	4	zuan4	4
喊	small	han3	12	107	3.3336	han4	3	huo3	3
珊	small	shan1	9	177	2.8082	shan4	4	shuo1	4
筋	small	jin1	12	162	2.8621	jin4	3	jue1	3
倒	small	dao3	10	125	4.0758	dao4	3	dun3	3

item	PND Group	pinyin	Strokes	PND	logCHR	Tone-edit neighbor	Pinyin length (Tone-edit neighbor)	Constituent-edit neighbor	Pinyin length (Constituent-edit neighbor)
柔	small	rou2	9	79	3.1881	rou4	3	ren2	3
章	small	zhang1	11	184	3.5514	zhang3	5	xiang1	5
昏	small	hun1	8	94	3.273	hun2	3	hou1	3
倡	small	chang4	10	121	3.8038	chang1	5	sang4	4
典	small	dian3	8	97	4.5688	dian4	4	ding3	4
茬	small	cha2	9	135	3.1523	cha1	3	xia2	3
掺	small	chan1	11	162	2.933	chan3	4	chui1	4
劲	small	jin4	7	139	3.5834	jin1	3	xun4	3
冒	small	mao4	9	153	3.7581	mao2	3	miu4	3
尘	small	chen2	6	122	2.9894	chen4	4	chui2	4
前	small	qian2	9	126	2.9839	qian4	4	qing2	4
羞	small	xiu1	10	160	3.3296	xiu4	3	xue1	3
闲	small	xian2	7	112	3.3015	xian1	4	xing2	4
曹	small	cao2	11	104	2.9533	cao3	3	cen2	3
竭	small	jie2	14	115	2.917	jie4	3	xue2	3
卵	small	luan3	7	135	3.7843	luan4	4	ling3	4
秀	small	xiu4	7	151	3.7043	xiu1	3	xun4	3
边	small	bian1	5	119	4.491	bian4	4	bang1	4
原	small	yuan2	10	100	3.8573	yuan4	4	ying2	4
调	small	tiao4	10	138	4.1625	tiao2	4	tuan2	4
臣	small	chen2	6	122	2.6693	chen4	4	chui2	4
泵	small	beng4	9	119	3.2175	beng1	4	bian4	4
每	small	mei3	7	98	4.4519	mei2	3	min3	3
阵	small	zhen4	6	157	3.3854	zhen1	4	zhou4	4
厅	small	ting1	7	98	3.5587	ting4	4	tuan1	4
羡	small	xian4	12	156	3.4728	xian1	4	xing4	4
矛	small	mao2	5	158	2.8762	mao1	3	men2	3

item	PND Group	pinyin	Strokes	PND	logCHR	Tone-edit neighbor	Pinyin length (Tone-edit neighbor)	Constituent-edit neighbor	Pinyin length (Constituent-edit neighbor)
凯	small	kai3	8	51	3.8675	kai1	3	kua3	3
欠	small	qian4	4	129	3.4716	qian2	4	qing4	4
命	small	ming4	8	97	3.9749	ming2	4	miao4	4
更	small	geng1	7	143	4.549	geng3	4	guan1	4
随	small	sui2	11	59	4.0881	sui4	3	san2	3
采	small	cai3	8	39	3.4983	cai4	3	cen3	3
陪	small	pei2	10	108	3.8454	pei4	3	pin2	3
响	small	xiang3	9	91	2.9454	xiang4	5	chang3	5
反	small	fan3	4	108	4.2019	fan4	3	fei3	3
听	small	ting1	7	98	4.9115	ting2	4	tuan1	4
唆	small	suo1	10	85	3.1875	suo3	3	sou1	3
少	small	shao3	4	125	4.4513	shao1	4	shua3	4
跷	small	qiao1	13	173	3.3263	qiao3	4	qing1	4
詹	small	zhan1	13	206	3.2744	zhan4	4	zhuo1	4

Note:All stimuli and primes (including Pinyin, stroke count, PND, and logCHR) were selected from the word neighborhood database created by Li et al. [2024]. The calculation of Pinyin length followed the method described by Neergaard et al. [2022].
