# Supplementary material for: Phonological Neighborhood Density and Type Modulate Visual Recognition of Mandarin Chinese: Evidence from Monosyllabic Words
Source: Brain Sci. 2025 Dec 2;15(12):1304. doi: 10.3390/brainsci15121304 (PMC12730931; doi:10.3390/brainsci15121304)
Supplement: Supplementary file 1 [file brainsci-15-01304-s001.zip › Supplementary Table S3: Full List of Pseudocharacter Stimulus.pdf]

Supplementary Methods: Pseudocharacter Stimuli

To maintain experimental balance, 120 Chinese pseudocharacters were constructed by recombining legal sublexical components from the target characters. These pseudocharacters met the following criteria:

(1) strict adherence to Chinese orthographic combination rules;

(2) matched structural topology with target characters (e.g., left-right, top-bottom, or single-unit configurations);

(3) comparable visual complexity

(4) absence of semantic or phonological validity

All pseudocharacters were generated using professional graphic tools to ensure stroke clarity and visual consistency. The complete pseudocharacter list containing identifying numbers, character images, and stroke counts is provided in the subsequent table of this supplementary material.

Stimulus ID	Pseudocharacter	Stroke Count	Stimulus ID	Pseudocharacter	Stroke Count
1	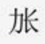	6	61	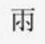	6
2	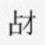	8	62	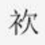	9
3	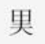	9	63	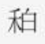	10
4	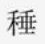	13	64	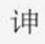	7
5	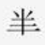	6	65	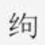	8
6	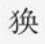	10	66	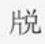	11
7	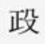	9	67	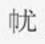	7
8	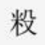	10	68	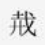	9

Stimulus ID	Pseudocharacter	Stroke Count	Stimulus ID	Pseudocharacter	Stroke Count
9	⿰舌阝	7	69	⿰立乍	10
10	⿱加云	9	70	⿰阝冬	7
11	专	3	71	⿰冫羊	8
12	亇	4	72	⿱穴几	7
13	⿸疒几	7	73	⿰衤囟	10
14	侹	8	74	⿸广夫	8
15	⿰丬召	8	75	⿱穴弃	12
16	⿰冫昔	10	76	⿰山咸	12
17	善	12	77	⿰衤册	9
18	⿰工半	7	78	⿸尸肋	12
19	⿰车不	8	79	⿰冫到	10
20	⿱山方	7	80	⿱丕灬	9
21	⿰山刂	10	81	⿱大早	11

Stimulus ID	Pseudocharacter	Stroke Count	Stimulus ID	Pseudocharacter	Stroke Count
22	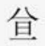	9	82	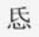	8
23	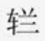	9	83	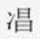	10
24	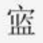	13	84	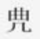	8
25	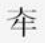	7	85	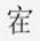	9
26	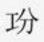	7	86	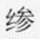	11
27	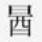	12	87	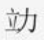	7
28	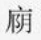	9	88	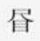	9
29	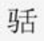	9	89	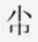	6
30	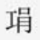	10	90	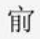	9
31	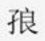	10	91	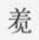	10
32	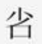	7	92	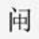	7
33	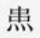	11	93	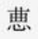	11
34	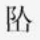	7	94	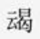	14

Stimulus ID	Pseudocharacter	Stroke Count	Stimulus ID	Pseudocharacter	Stroke Count
35	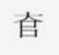	6	95	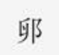	6
36	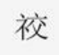	10	96	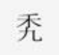	7
37	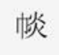	11	97	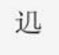	5
38	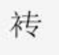	9	98	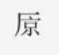	10
39	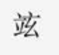	10	99	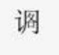	10
40	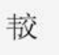	10	100	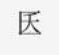	6
41	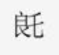	8	101	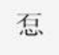	9
42	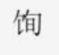	9	102	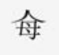	7
43	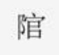	10	103	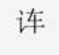	6
44	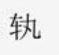	7	104	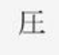	7
45	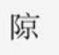	10	105	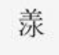	12
46	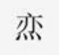	10	106	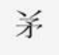	6
47	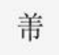	9	107	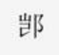	8

Stimulus ID	Pseudocharacter	Stroke Count	Stimulus ID	Pseudocharacter	Stroke Count
48	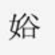	10	108	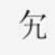	4
49	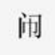	6	109	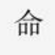	9
50	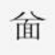	9	110	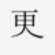	7
51	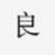	6	111	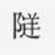	11
52	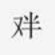	7	112	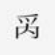	8
53	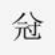	9	113	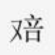	10
54	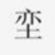	9	114	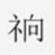	9
55	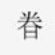	9	115	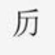	4
56	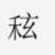	10	116	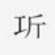	7
57	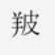	10	117	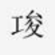	10
58	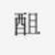	12	118	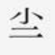	4
59	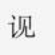	6	119	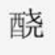	13
60	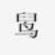	11	120	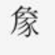	13
